# Two web-based dynamic prediction models for the diagnosis and prognosis of gastric cancer with bone metastases: evidence from the SEER database

**DOI:** 10.3389/fendo.2023.1136089

**Published:** 2023-05-24

**Authors:** Bo Liu, Kangpeng Li, Rui Ma, Qiang Zhang

**Affiliations:** Department of Orthopedics, Beijing Ditan Hospital, Capital Medical University, Beijing, China

**Keywords:** risk factors, gastric cancer, bone metastases, SEER, nomogram

## Abstract

**Purpose:**

Our aim was to identify the clinical characteristics and develop and validate diagnostic and prognostic web-based dynamic prediction models for gastric cancer (GC) with bone metastasis (BM) using the SEER database.

**Method:**

Our study retrospectively analyzed and extracted the clinical data of patients aged 18-85 years who were diagnosed with gastric cancer between 2010 and 2015 in the SEER database. We randomly divided all patients into a training set and a validation set according to the ratio of 7 to 3. Independent factors were identified using logistic regression and Cox regression analyses. Furthermore, we developed and validated two web-based clinical prediction models. We evaluated the prediction models using the C-index, ROC, calibration curve, and DCA.

**Result:**

A total of 23,156 patients with gastric cancer were included in this study, of whom 975 developed bone metastases. Age, site, grade, T stage, N stage, brain metastasis, liver metastasis, and lung metastasis were identified as independent risk factors for the development of BM in GC patients. T stage, surgery, and chemotherapy were identified as independent prognostic factors for GC with BM. The AUCs of the diagnostic nomogram were 0.79 and 0.81 in the training and test sets, respectively. The AUCs of the prognostic nomogram at 6, 9, and 12 months were 0.93, 0.86, 0.78, and 0.65, 0.69, 0.70 in the training and test sets, respectively. The calibration curve and DCA showed good performance of the nomogram.

**Conclusions:**

We established two web-based dynamic prediction models in our study. It could be used to predict the risk score and overall survival time of developing bone metastasis in patients with gastric cancer. In addition, we also hope that these two web-based applications will help physicians comprehensively manage gastric cancer patients with bone metastases.

## Introduction

Gastric cancer (GC) is a common and highly malignant type of cancer that affects the digestive system and is the second leading cause of cancer death worldwide (1). It is a relatively common cancer worldwide and is particularly prevalent in Asia, especially in countries such as Japan, Korea, and China. In contrast, it is relatively rare in North America and Western Europe (2). Certain genetic mutations can increase a person’s risk of developing gastric cancer. These mutations are more common in certain populations, such as those with a family history of gastric cancer or those with a history of certain inherited syndromes. Lifestyle factors such as smoking, heavy alcohol consumption, and a diet high in salted or smoked foods have been linked to an increased risk of gastric cancer. Infection with the bacterium *Helicobacter pylori* is a known risk factor for gastric cancer (3). This bacterium is very common and is often acquired in childhood. In addition, gastric cancer is characterized by rapid growth, high invasiveness, easy recurrence, and poor prognosis, among which invasiveness and easy metastasis are its main features (4, 5). Gastric cancer often spreads to the liver, lungs, and brain, but bone metastasis (BM) is relatively rare, with an incidence ranging from 1.2% to 1.4% (6) and 15.9% to 17.6% detected at autopsy (7).

GC patients with BM often experience a poor prognosis and shortened survival time, as cancer cells have spread to the bone and are often accompanied by metastasis to other organs (8). This can lead to bone pain, significantly affecting the patient’s quality of life (9, 10). Notably, bone metastasis can cause a range of symptoms, including pain, fractures, and spinal cord compression. Recent studies have identified specific molecular pathways that may be involved in the spread of gastric cancer to the bones. For example, certain proteins produced by gastric cancer cells, such as CXCR4 and RANKL, may interact with proteins in bone tissue, promoting the growth of new tumors (11). Early diagnosis is crucial for judging whether gastric cancer patients will develop bone metastases. The early implementation of active preventive and therapeutic measures greatly improves the survival time of gastric cancer patients (12). Therefore, it is important to identify the independent risk factors for the development of BM in GC patients and the risk factors for the prognosis of patients with GC and BM.

In recent years, an increasing number of clinical prediction models have been used in clinical practice. Among them, the nomogram presents data in an easily understandable graphical format based on the results of multiple regression analysis. Nomograms are also often used in medical research on cancer prognosis and promote personalized treatment of the disease by clinical physicians (13). However, to our knowledge, no researchers have yet calculated the risk score for bone metastasis in gastric cancer patients using a web-based prediction model, nor have they established a prognostic model to predict overall survival in GC patients with BM. Therefore, the aim of this study was to use the large sample size in the SEER database to establish and validate diagnostic and prognostic dynamic models (based on the web application) for gastric cancer patients with bone metastasis to fill this gap, and we hope that these two dynamic models can provide a basis for clinical diagnosis and treatment.

## Materials and methods

### Study design and data selection

The data for this study were downloaded from the Surveillance, Epidemiology, and End Results (SEER) Program (www.seer.cancer.gov) SEER*Stat Database software (version 8.4.0.1 released on 5/17/2022). The specific database we chose was the Incidence - SEER Research Plus Data, 17 Registries, Nov 2021 Sub (2000–2019) - Linked to County Attributes - Time-Dependent (1990-2019) Income/Rurality, 1969-2020 Counties, National Cancer Institute, DCCPS, Surveillance Research Program, released April 2022 and based on the November 2021 submission. The ethics committee of Beijing Ditan Hospital, Capital Medical University, approved the study. The study did not require medical ethical review or informed consent from patients. The ethics committee waived the requirement for informed consent from all patients.

**Figure 1:**
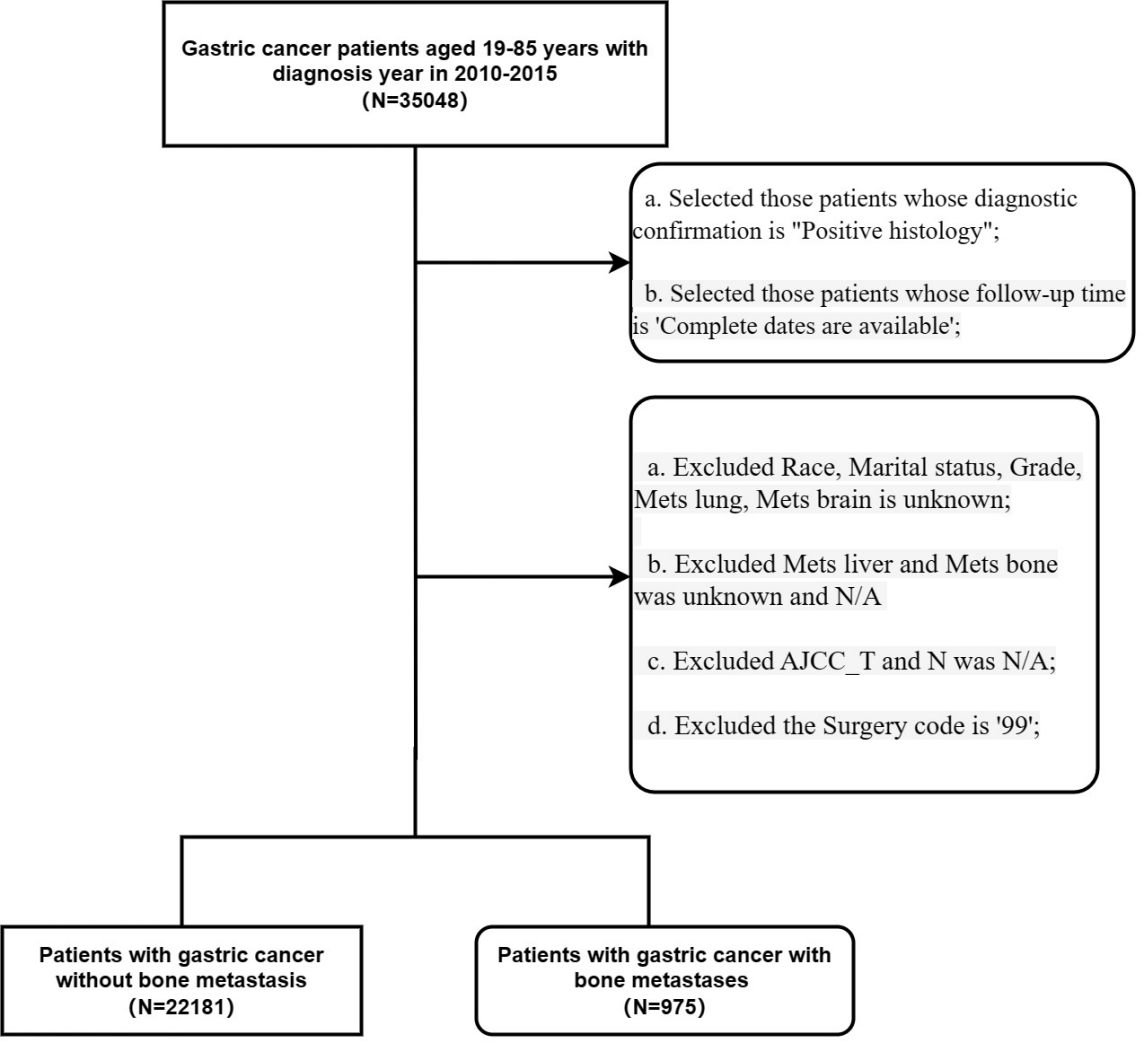
Flow chart of this study.

The inclusion criteria for the study were as follows: (1) histological diagnosis of gastric cancer; (2) the year of diagnosis was from 2010 to 2015; and (3) patients aged between 19 and 85 years old whose follow-up dates were available. The exclusion criteria were as follows: Selected those patients whose diagnostic confirmation was ‘Positive histology’ (2); Selected those patients whose follow-up time was ‘Complete dates are available’ (3); Excluded Race, marital status, grade, lung metastasis status, or brain metastasis status was unknown (2); patients whose liver or bone metastasis status was unknown or ‘N/A’ (3); patients for whom AJCC_T and N were ‘N/A’; and (4) patients with a surgery code of ‘99’ (**Figure 1**).

### Patient variables and outcome variables

Age, sex, ethnicity, primary site, grade, T stage, N stage, liver metastasis, lung metastasis, brain metastasis, and marital status were collected to identify risk factors for the development of BM in GC patients. The age of the patients was divided into two groups: <60 years and ≥60 years. The outcome variable of the diagnostic model was defined as whether patients with gastric cancer developed bone metastases.

The specific metastatic data (e.g., liver, lung, and brain metastasis) and treatment data (e.g., surgery, radiotherapy, and chemotherapy) were collected to form a new cohort, which was used to identify prognostic factors to establish a prognostic nomogram. The follow-up time (months) and survival status were defined as outcome variables in the prognostic model. The primary endpoint of the study was overall survival (OS), which was defined as the time from diagnosis to death due to any cause (14).

### Statistical analysis

R software (version 4.1.3) was used for statistical analyses in this study. The chi-square test was used to compare variables between the training set and test set, and a p value of less than 0.05 (bilateral) was considered statistically significant. The cohort was randomly divided into a training set (70%) and a test set (30%), with the training set used to establish the nomogram and the test set used to validate the models. Univariate and multivariate logistic regression analyses were used to identify independent diagnostic risk factors for GC with BM. The ‘rms’ and ‘regplot’ packages in R were used to create a diagnostic prediction model. In addition, a web-based dynamic model was developed based on the nomogram using the ‘Dynnom’ package. Similarly, univariate and multivariate Cox regression analyses were used to identify independent prognostic factors for GC metastasis. The ‘survival’ package in R was used to create prognostic nomograms. Similarly, we then developed a web-based prognostic model that predicted overall survival time by using the ‘Dynnom’ package. The two dynamic model applications above were released based on this website https://www.shinyapps.io/ The ROC and AUC were generated to evaluate the discrimination of the nomograms. Finally, calibration curve and decision curve analyses (DCA) were performed to evaluate the prediction ability of the nomograms.

## Result

### Demographic characteristics of the population and risk factors for BM in GC

Ultimately, our research included 22,181 participants who met the criteria, and 975 GC patients developed BM. We randomly divided all participants into a training set (16,212) and a test set (6,980). Among them, 975 (4.39%) patients developed BM of GC, while 22,181 did not (**Table 1**). We used multivariate logistic regression analysis to identify variables associated with the development of BM in GC patients and found that 8 factors were significantly associated: age (≥ 60 years), grade, primary site, higher T stage, higher N stage, brain metastasis, liver metastasis, and lung metastasis (**Table 2**, all p value < 0.05).

**Table 1:** Clinical characteristics of patients with bone metastasis of gastric cancer in train set and test set.

Group	Overall			Train Set			Test Set		
Mets_bone	No	Yes	P-value	No	Yes	P-value	No	Yes	P-value
N	N=22181	N=975		N=15544	N=668		N=6637	N=307	
Age	65.3 (12.6)	62.0 (13.2)	<0.001	65.3 (12.6)	62.0 (13.1)	<0.001	65.2 (12.6)	61.9 (13.5)	<0.001
Sex:			0.006			0.017			0.202
Male	14114 (63.6%)	663 (68.0%)		9799 (63.0%)	452 (67.7%)		4315 (65.0%)	211 (68.7%)	
Female	8067 (36.4%)	312 (32.0%)		5745 (37.0%)	216 (32.3%)		2322 (35.0%)	96 (31.3%)	
Race:			0.002			0.03			0.012
White	15896 (71.7%)	739 (75.8%)		11104 (71.4%)	494 (74.0%)		4792 (72.2%)	245 (79.8%)	
Black	2884 (13.0%)	90 (9.23%)		2032 (13.1%)	64 (9.58%)		852 (12.8%)	26 (8.47%)	
Other	3401 (15.3%)	146 (15.0%)		2408 (15.5%)	110 (16.5%)		993 (15.0%)	36 (11.7%)	
Marital_status:			0.382			0.474			0.641
No	8484 (38.2%)	387 (39.7%)		6010 (38.7%)	268 (40.1%)		2474 (37.3%)	119 (38.8%)	
Yes	13697 (61.8%)	588 (60.3%)		9534 (61.3%)	400 (59.9%)		4163 (62.7%)	188 (61.2%)	
Site:			<0.001			<0.001			<0.001
Other	11094 (50.0%)	485 (49.7%)		7762 (49.9%)	331 (49.6%)		3332 (50.2%)	154 (50.2%)	
Cardia	7183 (32.4%)	401 (41.1%)		5058 (32.5%)	279 (41.8%)		2125 (32.0%)	122 (39.7%)	
Gastric antrum	3904 (17.6%)	89 (9.13%)		2724 (17.5%)	58 (8.68%)		1180 (17.8%)	31 (10.1%)	
Grade:			<0.001			<0.001			<0.001
I	2419 (10.9%)	13 (1.33%)		1713 (11.0%)	8 (1.20%)		706 (10.6%)	5 (1.63%)	
II	5920 (26.7%)	154 (15.8%)		4148 (26.7%)	119 (17.8%)		1772 (26.7%)	35 (11.4%)	
III	13275 (59.8%)	789 (80.9%)		9303 (59.8%)	528 (79.0%)		3972 (59.8%)	261 (85.0%)	
IV	567 (2.56%)	19 (1.95%)		380 (2.44%)	13 (1.95%)		187 (2.82%)	6 (1.95%)	
AJCC_T:			<0.001			<0.001			<0.001
T1	5512 (24.9%)	187 (19.2%)		3915 (25.2%)	128 (19.2%)		1597 (24.1%)	59 (19.2%)	
T2	2642 (11.9%)	39 (4.00%)		1831 (11.8%)	27 (4.04%)		811 (12.2%)	12 (3.91%)	
T3	6074 (27.4%)	136 (13.9%)		4227 (27.2%)	100 (15.0%)		1847 (27.8%)	36 (11.7%)	
T4	7953 (35.9%)	613 (62.9%)		5571 (35.8%)	413 (61.8%)		2382 (35.9%)	200 (65.1%)	
AJCC_N:			<0.001			<0.001			0.001
N1	16505 (74.4%)	725 (74.4%)		11581 (74.5%)	496 (74.3%)		4924 (74.2%)	229 (74.6%)	
N2	2124 (9.58%)	48 (4.92%)		1498 (9.64%)	36 (5.39%)		626 (9.43%)	12 (3.91%)	
N3	3552 (16.0%)	202 (20.7%)		2465 (15.9%)	136 (20.4%)		1087 (16.4%)	66 (21.5%)	
Mets_brain:			<0.001			<0.001			<0.001
No	22078 (99.5%)	938 (96.2%)		15478 (99.6%)	640 (95.8%)		6600 (99.4%)	298 (97.1%)	
Yes	103 (0.46%)	37 (3.79%)		66 (0.42%)	28 (4.19%)		37 (0.56%)	9 (2.93%)	
Mets_liver:			<0.001			<0.001			<0.001
No	19162 (86.4%)	622 (63.8%)		13428 (86.4%)	434 (65.0%)		5734 (86.4%)	188 (61.2%)	
Yes	3019 (13.6%)	353 (36.2%)		2116 (13.6%)	234 (35.0%)		903 (13.6%)	119 (38.8%)	
Mets_lung:			<0.001			<0.001			<0.001
No	21305 (96.1%)	737 (75.6%)		14943 (96.1%)	508 (76.0%)		6362 (95.9%)	229 (74.6%)	
Yes	876 (3.95%)	238 (24.4%)		601 (3.87%)	160 (24.0%)		275 (4.14%)	78 (25.4%)	

**Table 2:** Univariate and multivariate logistics analysis of bone metastasis in gastric cancer patients.

Characteristics	Univariate Analysis			Multivariate Analysis		
	OR	CI	P-value	OR	CI	P-value
Age	0.98	0.98-0.99	<0.001	0.98	0.98-0.99	<0.001
AJCC_T						
T1	Ref	Ref		Ref	Ref	
T2	0.45	0.3-0.69	<0.001	0.53	0.35-0.81	0.003
T3	0.72	0.56-0.94	0.017	0.69	0.52-0.91	0.008
T4	2.27	1.85-2.78	<0.001	1.64	1.32-2.04	<0.001
Grade						
I	Ref	Ref		Ref	Ref	
II	6.14	3-12.59	<0.001	4.45	2.15-9.19	<0.001
III	12.15	6.04-24.46	<0.001	9.25	4.57-18.73	<0.001
IV	7.33	3.02-17.79	<0.001	5.2	2.1-12.87	<0.001
Marital_status						
No	Ref	Ref				
Yes	0.94	0.8-1.1	0.45			
Mets_brain						
No	Ref	Ref		Ref	Ref	
Yes	10.26	6.55-16.08	<0.001	4.2	2.54-6.94	<0.001
Mets_liver						
No	Ref	Ref		Ref	Ref	
Yes	3.42	2.9-4.04	<0.001	1.75	1.44-2.11	<0.001
Mets_lung						
No	Ref	Ref		Ref	Ref	
Yes	7.83	6.44-9.52	<0.001	4.29	3.45-5.34	<0.001
AJCC_N						
N1	Ref	Ref		Ref	Ref	
N2	0.56	0.4-0.79	0.001	0.62	0.44-0.88	0.008
N3	1.29	1.06-1.57	0.011	0.89	0.72-1.09	0.252
Race						
White	Ref	Ref		Ref	Ref	
Black	0.71	0.54-0.92	0.011	0.78	0.59-1.03	0.081
Other	1.03	0.83-1.27	0.806	1.21	0.97-1.51	0.097
Sex						
Male	Ref	Ref		Ref	Ref	
Female	0.82	0.69-0.96	0.015	0.93	0.78-1.11	0.409
Site						
Other	Ref	Ref		Ref	Ref	
Cardia	1.29	1.1-1.52	0.002	1.22	1.02-1.47	0.032
Gastric antrum	0.5	0.38-0.66	<0.001	0.51	0.38-0.67	<0.001

### The diagnostic nomogram and web-based application for developing BM in GC

**Figure 2:**
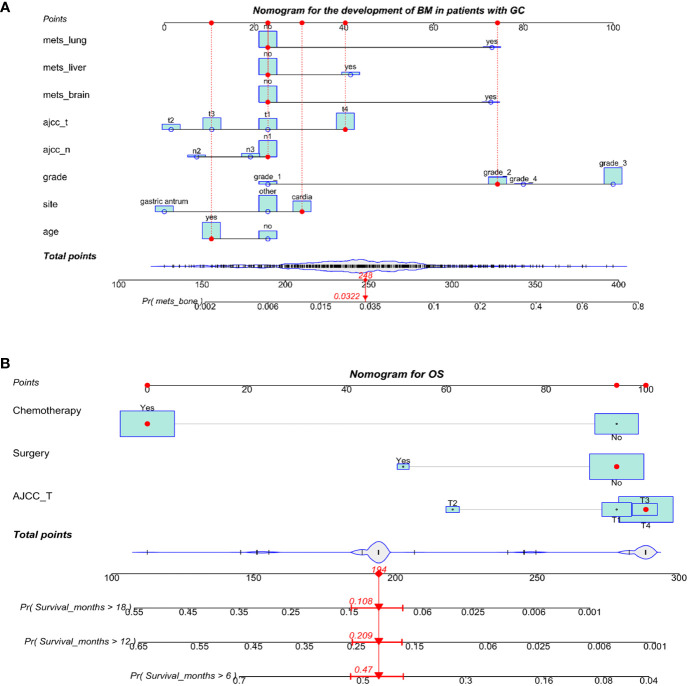
The diagnosis (**A**) and prognosis (**B**) nomograms for predicting bone metastasis in patients with gastric cancer.

**Figure 3:**
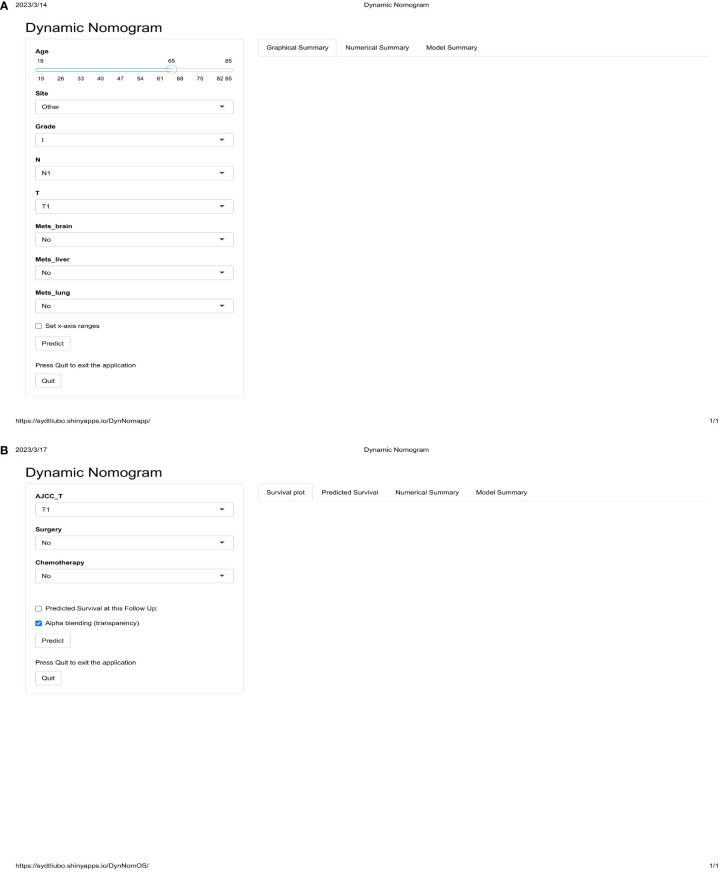
The operation interface of two web-based dynamic diagnostic **(A)** and prognostic **(B)** prediction models for gastric cancer patients with bone metastases.

We used eight independent risk factors for the development of BM in GC to create a diagnostic nomogram for assessing the risk score of developing BM in GC patients (**Figure 2A**). In addition, based on the established nomogram, we further developed a dynamic web-based application. This can be accessed by clicking on a hyperlink (**Figure 3A**, https://sydtliubo.shinyapps.io/DynNomapp/).

**Figure 4:**
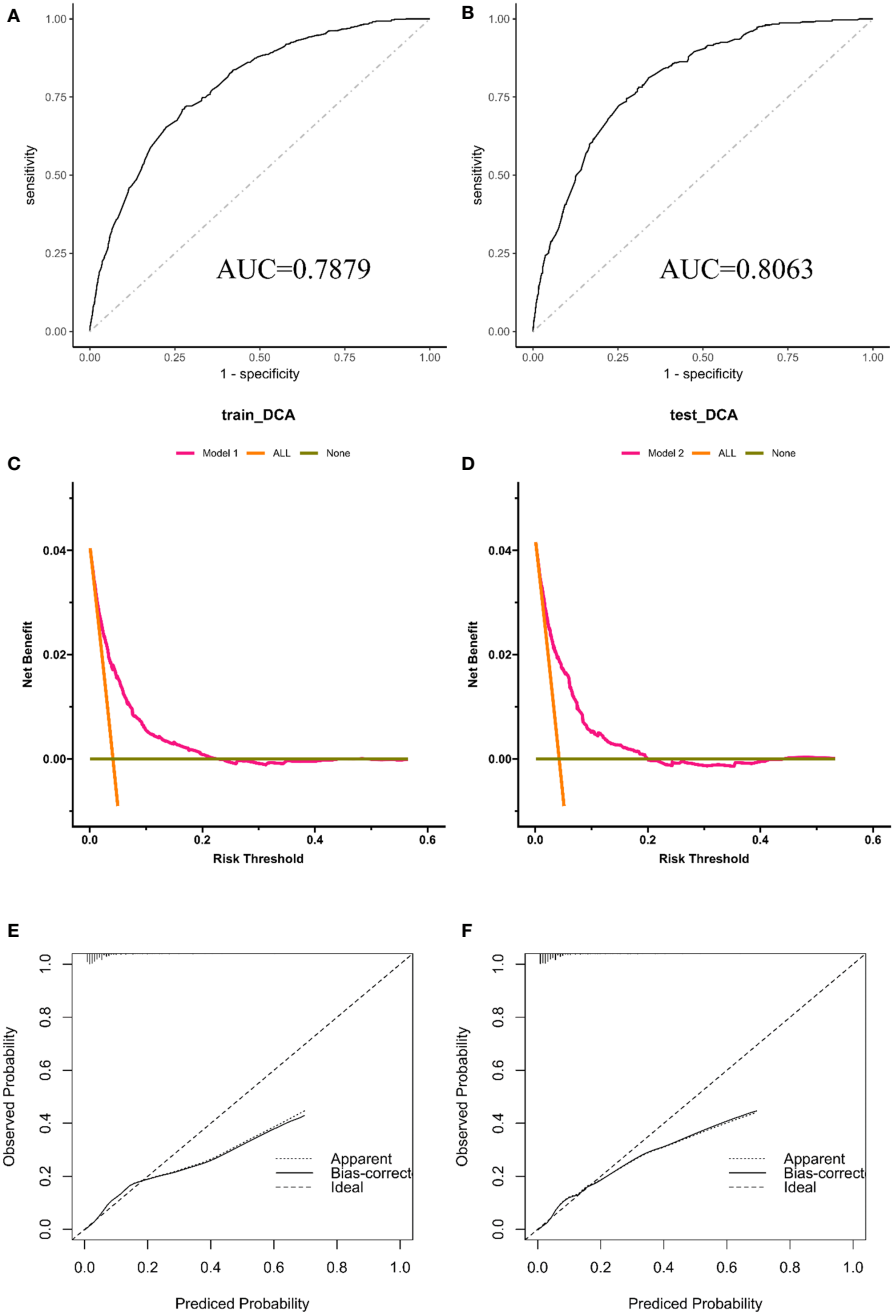
The receiver operating characteristic curve **(A)**, calibration curve **(C)** and decision curve analysis **(E)** in the training set. The receiver operating characteristic curve **(B)**, calibration curve **(D)**, and decision curve analysis **(F)** in the testing set.

The AUC of the nomogram was 0.788 in the test group and 0.810 in the validation group (**Figures 4A**, **B**), indicating a high predictive value for the nomogram. We also plotted the calibration curve and DCA for the nomogram in both the training and test sets, which showed that the nomogram is a good diagnostic tool for predicting BM in GC (**Figures 4C–F**).

### Prognostic factors for gastric cancer patients with bone metastases

We analyzed 975 patients who developed BM from GC to identify prognostic factors. We randomly assigned 684 of these patients to the training set and the remaining 291 patients to the test set. The chi-square test showed no significant difference between the training and test sets (**Table 3**). Univariate and multivariate Cox regression analyses showed that AJCC T stage (P=0.012), surgery (P<0.001), and chemotherapy (P<0.001) were independent prognostic factors for GC patients with BM (**Table 4**).

**Table 3:** Patient clinical characteristics of gastric cancer with bone metastases in the train set and test set.

Group	Overall			Train set			Test set		
Status	Alive	Death	P-value	Alive	Death	P-value	Alive	Death	P-value
N	N=29	N=946		N=21	N=663		N=8	N=283	
Age:			0.025			0.015			0.734
No	19 (65.5%)	405 (42.8%)		15 (71.4%)	281 (42.4%)		4 (50.0%)	124 (43.8%)	
Yes	10 (34.5%)	541 (57.2%)		6 (28.6%)	382 (57.6%)		4 (50.0%)	159 (56.2%)	
Sex:			0.929			1			0.716
Male	19 (65.5%)	644 (68.1%)		14 (66.7%)	452 (68.2%)		5 (62.5%)	192 (67.8%)	
Female	10 (34.5%)	302 (31.9%)		7 (33.3%)	211 (31.8%)		3 (37.5%)	91 (32.2%)	
Race:			0.004			0.015			0.26
White	17 (58.6%)	722 (76.3%)		12 (57.1%)	516 (77.8%)		5 (62.5%)	206 (72.8%)	
Black	1 (3.45%)	89 (9.41%)		1 (4.76%)	58 (8.75%)		0 (0.00%)	31 (11.0%)	
Other	11 (37.9%)	135 (14.3%)		8 (38.1%)	89 (13.4%)		3 (37.5%)	46 (16.3%)	
Marital_status:			0.124			0.145			0.718
No	16 (55.2%)	371 (39.2%)		12 (57.1%)	258 (38.9%)		4 (50.0%)	113 (39.9%)	
Yes	13 (44.8%)	575 (60.8%)		9 (42.9%)	405 (61.1%)		4 (50.0%)	170 (60.1%)	
Site:			0.126			0.112			0.394
Other	18 (62.1%)	467 (49.4%)		12 (57.1%)	331 (49.9%)		6 (75.0%)	136 (48.1%)	
Cardia	7 (24.1%)	394 (41.6%)		5 (23.8%)	274 (41.3%)		2 (25.0%)	120 (42.4%)	
Gastric antrum	4 (13.8%)	85 (8.99%)		4 (19.0%)	58 (8.75%)		0 (0.00%)	27 (9.54%)	
Grade:			0.649			0.771			0.234
I	0 (0.00%)	13 (1.37%)		0 (0.00%)	7 (1.06%)		0 (0.00%)	6 (2.12%)	
II	3 (10.3%)	151 (16.0%)		2 (9.52%)	108 (16.3%)		1 (12.5%)	43 (15.2%)	
III	25 (86.2%)	764 (80.8%)		19 (90.5%)	534 (80.5%)		6 (75.0%)	230 (81.3%)	
IV	1 (3.45%)	18 (1.90%)		0 (0.00%)	14 (2.11%)		1 (12.5%)	4 (1.41%)	
AJCC_T:			0.715			0.561			0.621
T1	6 (20.7%)	181 (19.1%)		4 (19.0%)	117 (17.6%)		2 (25.0%)	64 (22.6%)	
T2	2 (6.90%)	37 (3.91%)		2 (9.52%)	29 (4.37%)		0 (0.00%)	8 (2.83%)	
T3	4 (13.8%)	132 (14.0%)		2 (9.52%)	91 (13.7%)		2 (25.0%)	41 (14.5%)	
T4	17 (58.6%)	596 (63.0%)		13 (61.9%)	426 (64.3%)		4 (50.0%)	170 (60.1%)	
N:			0.345			0.149			1
N1	25 (86.2%)	700 (74.0%)		19 (90.5%)	498 (75.1%)		6 (75.0%)	202 (71.4%)	
N2	1 (3.45%)	47 (4.97%)		1 (4.76%)	27 (4.07%)		0 (0.00%)	20 (7.07%)	
N3	3 (10.3%)	199 (21.0%)		1 (4.76%)	138 (20.8%)		2 (25.0%)	61 (21.6%)	
Surgery:			1			0.616			1
No	28 (96.6%)	907 (95.9%)		20 (95.2%)	634 (95.6%)		8 (100%)	273 (96.5%)	
Yes	1 (3.45%)	39 (4.12%)		1 (4.76%)	29 (4.37%)		0 (0.00%)	10 (3.53%)	
Radiation:			0.538			0.142			0.254
No	22 (75.9%)	650 (68.7%)		18 (85.7%)	452 (68.2%)		4 (50.0%)	198 (70.0%)	
Yes	7 (24.1%)	296 (31.3%)		3 (14.3%)	211 (31.8%)		4 (50.0%)	85 (30.0%)	
Chemotherapy:			0.902			0.573			0.715
No	12 (41.4%)	364 (38.5%)		10 (47.6%)	259 (39.1%)		2 (25.0%)	105 (37.1%)	
Yes	17 (58.6%)	582 (61.5%)		11 (52.4%)	404 (60.9%)		6 (75.0%)	178 (62.9%)	
Mets_brain:			1			1			0.309
No	28 (96.6%)	910 (96.2%)		21 (100%)	639 (96.4%)		7 (87.5%)	271 (95.8%)	
Yes	1 (3.45%)	36 (3.81%)		0 (0.00%)	24 (3.62%)		1 (12.5%)	12 (4.24%)	
Mets_liver:			0.433			0.33			1
No	21 (72.4%)	601 (63.5%)		16 (76.2%)	420 (63.3%)		5 (62.5%)	181 (64.0%)	
Yes	8 (27.6%)	345 (36.5%)		5 (23.8%)	243 (36.7%)		3 (37.5%)	102 (36.0%)	
Mets_lung:			1			0.793			0.429
No	22 (75.9%)	715 (75.6%)		17 (81.0%)	504 (76.0%)		5 (62.5%)	211 (74.6%)	
Yes	7 (24.1%)	231 (24.4%)		4 (19.0%)	159 (24.0%)		3 (37.5%)	72 (25.4%)	
Survival_months	26.1 (31.4)	6.20 (8.10)	0.002	29.0 (33.3)	6.30 (8.29)	0.005	18.8 (26.0)	5.98 (7.64)	0.208

**Table 4:** Univariate and multivariate cox analysis of OS in patients with bone metastases from gastric cancer.

	Univariate Analysis			Multivariate Analysis		
	HR	95%CI	P-value	HR	95%CI	P-value
Age >60						
No	ref	ref				
Yes	1.114	0.980 -1.268	0.099			
Sex						
Male	ref	ref				
Female	1.031	0.899-1.182	0.659			
Marital_status						
No	ref	ref				
Yes	0.910	0.798-1.037	0.156			
Site						
Other	ref	ref		ref	ref	
Cardia	0.845	0.739-0.966	0.014	0.914	0.797-1.050	0.203
Gastric antrum	1.083	0.859-1.364	0.501	1.155	0.915-1.4550	0.226
Grade						
I	ref	ref				
II	0.792	0.449-1.396	0.420			
III	1.014	0.586-1.754	0.962			
IV	1.021	0.500-2.083	0.955			
AJCC_T						
T1	ref	ref		ref	ref	
T2	0.666	0.467-0.9487	0.024	0.633	0.443-0.903	0.012
T3	0.848	0.677-1.0613	0.150	1.008	0.820-1.267	0.947
T4	1.094	0.926-1.2917	0.293	1.081	0.915-1.278	0.359
AJCC_N						
N1	ref	ref				
N2	0.866	0.644-1.164	0.342			
N3	1.165	0.995-1.364	0.058			
Surgery						
No	ref	ref		ref	ref	
Yes	0.658	0.477-0.9073	0.011	0.512	0.369-0.711	<0.001
Radiation						
No	ref	ref		ref	ref	
Yes	0.870	0.758-0.9976	0.046	0.967	0.840-1.114	0.644
Chemotherapy						
No	ref	ref		ref	ref	
Yes	0.280	0.244-0.322	<0.001	0.268	0.233-0.309	<0.001
Mets_brain						
No	ref	ref				
Yes	1.016	0.728-1.42	0.927			
Mets_liver						
No	ref	ref				
Yes	0.9989	0.875-1.141	0.986			
Mets_lung						
No	ref	ref				
Yes	1.093	0.942-1.268	0.242			

### The prognostic nomogram and web-based application for OS of GC with BM

We developed a prognostic nomogram based on three independent prognostic factors (**Figure 2B**). Similarly, based on the established prognostic model, we further developed a dynamic web-based application. This can be accessed by clicking on a hyperlink (**Figure 3B**, https://sydtliubo.shinyapps.io/DynNomOS/).

**Figure 5:**
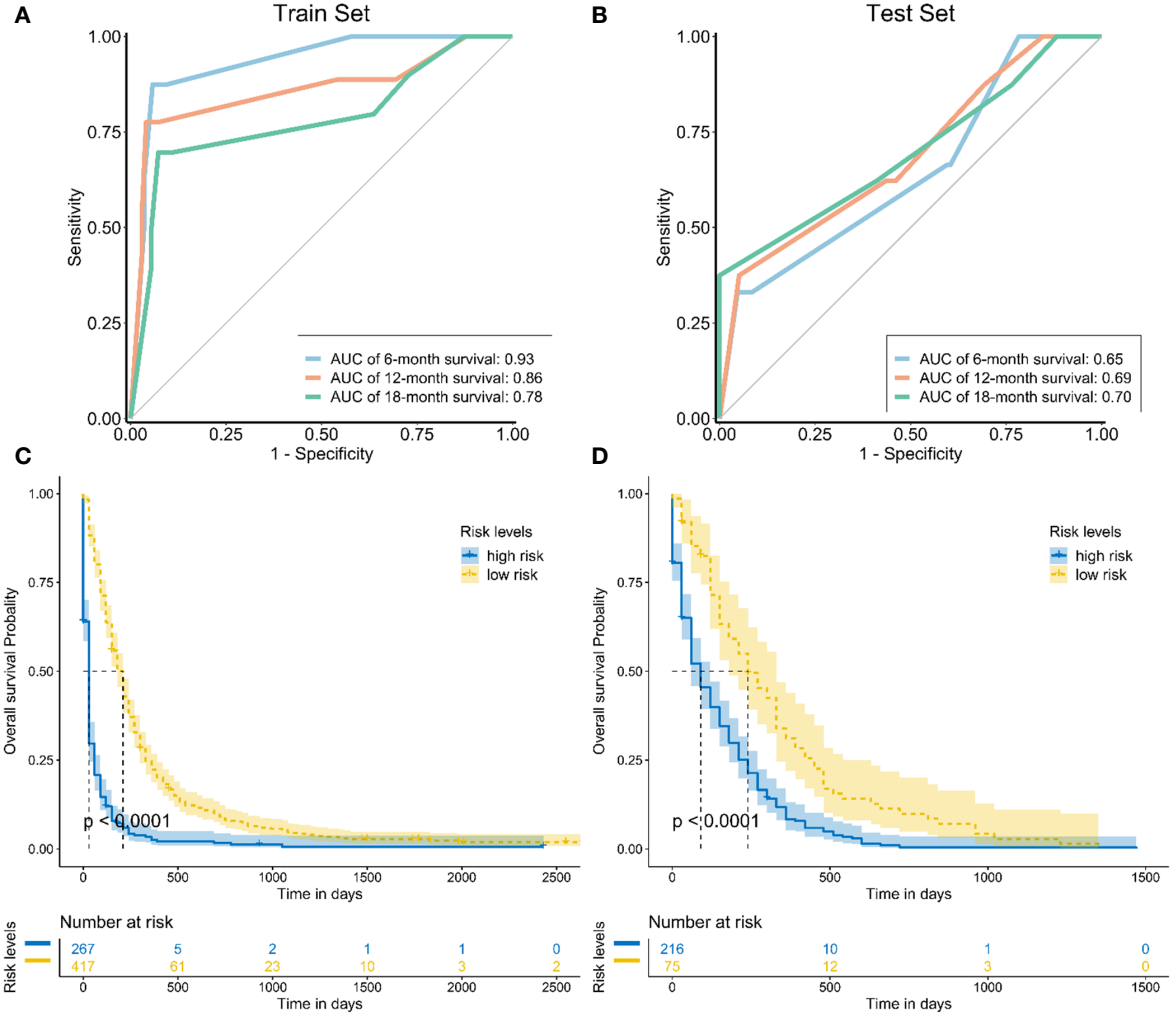
**(A)** The receiver operating curves at 6, 12, and 18 months in the training set; **(B)** The receiver operating curves at 6, 12, and 18 months in the test set; The Kaplan–Meier survival curves of three mortality risk subgroups in the training group **(C)** and testing group **(D)**.

**Figure 6:**
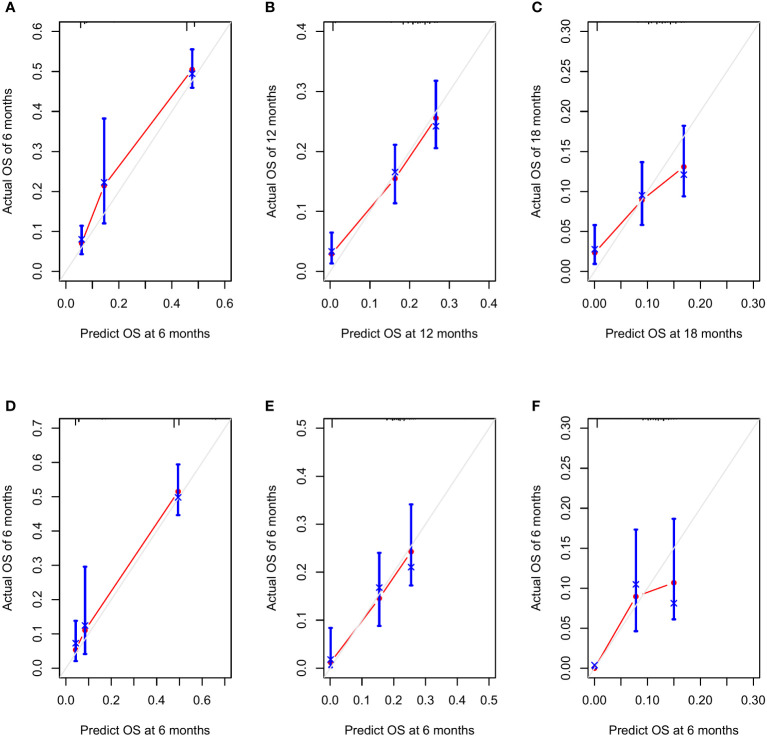
The calibration curves at 6 months **(A)**, 12 months **(B)**, and 18 months **(C)** in the training set and at 6 months **(D)**, 12 months **(E)**, and 18 months **(F)** in the testing set.

**Figure 7:**
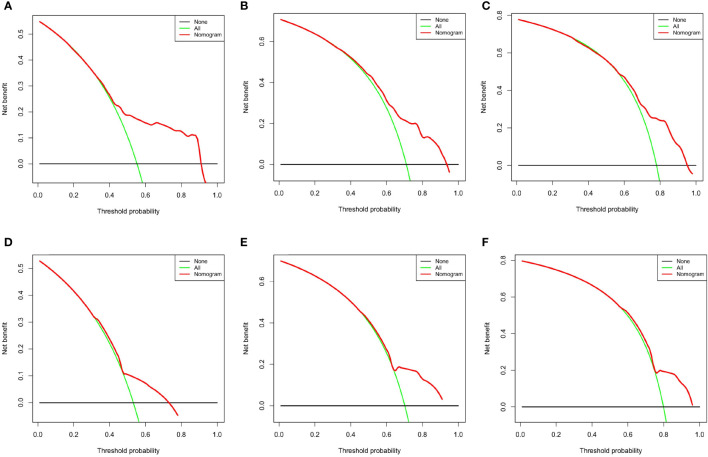
Decision curve analysis at 6 months **(A)**, 12 months **(B)**, and 18 months **(C)** in the training set and at 6 months **(D)**, 12 months **(E)**, and 18 months **(F)** in the testing set.

The areas under the curve (AUCs) at 6, 12, and 18 months were 0.93, 0.86 and 0.78 in the training set, respectively (**Figure 5A**). In the test set, the AUCs at 6, 12, and 18 months were 0.65, 0.69, and 0.70, respectively (**Figure 5B**). The calibration curves at 6, 12, and 18 months also showed that the nomogram predictions had good agreement with the actual outcomes (**Figures 6A–F**). Similarly, the DCA curves at 6, 12, and 18 months demonstrated that the nomogram had good predictive efficiency for the prognosis of patients with GC and BM (**Figures 7A–F**). The Kaplan–Meier survival analysis with log-rank test suggested a significant difference (P < 0.001) when comparing the survival curves for subgroups in both the training and test sets. Patients with high risk scores had a worse prognosis than those with low risk scores (**Figures 5C**, **D**).

## Discussion

To the best of our knowledge, there is still a lack of specific studies on the risk factors and survival analysis of gastric cancer patients with bone metastases. To better fill this gap, we used the SEER database with a larger sample size for analysis. We found it very inconvenient to use only graphical prediction models in clinical practice; therefore, we developed two web-based prediction models to predict the risk factors and survival status of gastric cancer patients with bone metastases. Clinicians only need to enter the population characteristics of each patient, and the two applications can quickly obtain their risk scores. We hope that these two web applications can be applied in the actual work of clinicians.

Gastric cancer is a cancer of the stomach characterized by high malignancy, rapid development, high invasiveness, easy recurrence, and poor prognosis. The prognosis for patients with GC becomes poor once bone metastases occur (15, 16). GC is currently the second leading cause of cancer-related death worldwide (17). Gastric cancer is a disease that disproportionately affects certain regions of the world. Approximately 70% of new cases occur in developing countries, particularly in eastern Asia. In contrast, the incidence of GC is lower in North America and northern Europe. These geographical imbalances in incidence and mortality are significant and highlight the need for targeted prevention and treatment efforts in regions with higher rates of GC (18, 19). The incidence of BM of GC in this study was 2.7%, and Turkoz et al. (7) found 176 bone metastases in 4617 patients with GC after investigating them, with an incidence of 3.8%. In the past, the prevailing opinion among investigators was that the incidence of BM in GC ranges from 0.8% to 2.1% (6, 20). GC cells have a tendency to spread to the bones if they invade the blood or lymphatic vessels within the stomach wall. Patients who have metastasis to abdominal lymph nodes, liver, and lungs, in particular, are at a higher risk of also developing BM simultaneously.

Our study found that the independent risk factors for the development of BM in GC were age, primary site, grade III, T stage, N stage, and brain, liver, and lung metastasis, but especially brain metastasis. Many previous studies have found that the primary gastric cancer lesions were located in the gastric antrum and body; in addition, patients with poorly differentiated tumors, deep local invasion, and lymph node metastasis are more likely to develop bone metastases (7, 8, 21). Qiu et al. (9) showed that compared with GC patients without distant metastasis, patients with distant metastasis were more likely to have BM. Liang et al. (22) also found that cardiac cancer, young age, low degree of differentiation, high N stage, and diffuse type were positively correlated with BM. We found that the factors mentioned above have been confirmed in previous studies. Overall, this is consistent with the conclusions of this study.

Our research shows that T stage, surgery and chemotherapy were independent prognostic factors for GC with BM. In other words, patients with primary tumors in the fundus and greater curvature of the stomach who underwent gastric surgery and who received chemotherapy had a lower risk of early death and longer survival. Liang et al. (22) also found that for GC patients with bone metastasis, the median survival time of the primary tumor operation group was longer than that of the nonoperation group. This is in accordance with the results of the present study. Similarly, although bone metastasis of GC is already an advanced stage, systemic chemotherapy should still be actively administered to prolong patient survival time (6, 7, 23), and the relationship between chemotherapy and its prognosis has also been demonstrated. In addition to systemic chemotherapy, the application of bisphosphonates also improves patient survival time (24). Using three independent prognostic factors, we developed a nomogram. The final results suggest that this nomogram may be a useful tool for identifying patients at high risk for poor outcomes.

At the molecular level, the expression of ALP, LDH, DIC, CEA, and CA 19-9 is associated with the development of BM and prognosis in GC (7, 25–27). When malignant tumors develop into BM, the normal bone metabolism mechanism is disrupted, which causes an increase in the rate of bone resorption and bone formation. The biomarkers vary in both the patient’s serum and in the urine (28, 29). However, these biomarkers are difficult to apply immediately to clinical decision-making. To date, no investigators have established diagnostic predictive models for the development of BM in GC and clinical predictive prognostic models for GC combined with BM. This means that it is not possible to combine all of the independent predictors associated with the occurrence of BM in GC. We could not identify the patient’s individualized risk of developing bone metastases on the whole.

The strength of our research is that we have successfully developed and validated two dynamic prediction models: the first one for predicting the risk score of BM in newly diagnosed GC patients and another for predicting the prognosis of GC patients with BM. Furthermore, we developed two applications of web-based predictive models. They will be put into practice by clinical orthopedic surgeons. The results showed that these nomograms have higher discriminant power than any single predictor, indicating the value of a comprehensive prediction model for more accurate individual clinical decision-making and monitoring. The total score for each GC patient can be calculated using the data for various variables on the appropriate nomogram (30–32). Using the nomograms, it is easy to calculate the risk of BM in GC and guide further clinical management. Similarly, the prognostic risk of GC patients with BM can be determined using the prognostic nomogram.

There are several limitations to our study. First, the sample size of patients with BM from GC was relatively small, which may have introduced bias. In future studies, we should aim to increase the sample size to reduce this bias. Second, the SEER database did not include information on specific sites of BM, which can significantly affect the prognostic survival of patients. We can infer that patients with multiple and extensive bone metastases are likely to have shorter survival times. Third, our study was a retrospective study and therefore inherently prone to selection bias. Additionally, detailed treatment information was not available in the SEER database.

## Conclusions

We established and validated two prediction models using the SEER database. Furthermore, we developed two applications of web-based predictive models. We hope that they will be used by clinical orthopedic surgeons in practice. They could be used to predict the risk score of bone metastasis in patients with gastric cancer and to predict the overall survival time of patients with gastric cancer with bone metastasis.

## Data availability statement

Publicly available datasets were analyzed in this study. This data can be found here: https://seer.cancer.gov/.

## Author contributions

BL performed the data analysis and wrote the manuscript. KL contributed to data extraction; RM contributed to the literature search; QZ conceived and designed the study. All authors contributed to the article and approved the submitted version.
